# Astragaloside IV Alleviates DSS-Induced Ulcerative Colitis by Modulating Host–Gut Tryptophan Metabolism

**DOI:** 10.3390/foods15101644

**Published:** 2026-05-08

**Authors:** Hongxia Yuan, Zhijun Yang, Chunmei Wu, Xinyu Chen, Lili Peng, Yajie Liu, Xinyi Wang, Yuanbiao Qiao, Fan Yang, Rui Ge, Qingshan Li

**Affiliations:** 1 Shanxi Key Laboratory of Innovative Drug for the Treatment of Serious Diseases Basing on the Chronic Inflammation, College of Traditional Chinese Medicine and Food Engineering, Shanxi University of Chinese Medicine, Jinzhong 030619, China; yuanhongxia609@sxtcm.edu.cn (H.Y.); yangzhijun@sxtcm.edu.cn (Z.Y.);; 2 Medicinal Basic Research Innovation Center of Chronic Kidney Disease, Ministry of Education, Shanxi Medical University, Jinzhong 030619, China

**Keywords:** Astragaloside IV, ulcerative colitis, gut microbiota, tryptophan metabolism, AhR, NF-κB p65

## Abstract

Astragaloside IV (AS-IV), a principal bioactive constituent of the medicinal and edible herb *Radix astragali*, exerts protective effects against ulcerative colitis (UC). This study investigated its underlying mechanisms in dextran sulfate sodium (DSS)-induced colitis using 16S rRNA sequencing, untargeted fecal metabolomics, and label-free proteomics. AS-IV intervention remodeled intestinal microbiota composition by markedly increasing *Akkermansia* abundance. Fecal metabolomic analysis revealed enhanced tryptophan (Trp) metabolism and elevated levels of kynurenic acid, 5-hydroxyindoleacetic acid and indole-3-acetic acid, which were significantly positively correlated with *Akkermansia* abundance. Proteomic analysis further identified Trp metabolism as a key pathway. Indoleamine 2,3-dioxygenase 1 (IDO1) and dopa decarboxylase (DDC) were recognized as differentially expressed proteins in colonic tissues. AS-IV ameliorated colitis by downregulating IDO1 expression, while upregulating the expression of tryptophan hydroxylase 1 (TPH1), DDC, monoamine oxidase A (MAO-A), and the aryl hydrocarbon receptor (AhR), as well as inhibiting NF-κB p65 phosphorylation. Collectively, these findings indicate that AS-IV enhances intestinal barrier function and mitigates colonic inflammation in DSS-induced UC. These beneficial effects are associated with the regulation of host–gut Trp metabolism, altered AhR expression, and suppressed NF-κB p65 activation. This study underscores the potential of AS-IV as a candidate functional food ingredient for the management of UC.

## 1. Introduction

Ulcerative colitis (UC) is classified as a subtype of inflammatory bowel disease (IBD), and it presents chronic, multifactorial inflammation primarily affecting the rectum and colon. The predominant clinical manifestations in individuals with UC include abdominal pain and diarrhea, frequently accompanied by intestinal bleeding [1]. Recent epidemiological data indicate a global prevalence of approximately 5 million UC cases in 2023, and the disease exhibits a rising incidence trend [2]. The prevailing understanding of UC pathogenesis suggests that a complex interplay of environmental and host factors enhances susceptibility. Inflammatory initiation and progression are governed by several core pathological alterations, including impaired intestinal epithelial barrier, gut microbiota dysbiosis, and aberrant immune activation [3]. Clinical management predominantly focuses on symptom alleviation through pharmacological interventions, such as sulfasalazine. However, secondary treatment failures or severe adverse effects are common [4,5]. Accordingly, natural bioactive compounds, especially food-derived components, have attracted increasing interest. They are expected to relieve intestinal inflammation and UC-related symptoms with milder side effects.

Tryptophan (Trp), an essential amino acid, plays a significant role in regulating gut inflammation, preserving epithelial barrier integrity, and maintaining energy homeostasis [6,7]. Studies have shown that Trp metabolism is dysregulated in UC patients, along with obvious changes in Trp and its downstream metabolites [8]. Trp supplementation has been shown to ameliorate dextran sulfate sodium (DSS)-induced colitis models [9,10]. A growing number of studies confirm that gut microbiota is a core regulator of Trp metabolism and mediates its anti-inflammatory properties [10,11]. *Akkermansia* (AKK) is a key bacterium capable of Trp metabolism. Increasing its abundance can significantly alleviate intestinal inflammation and mucosal injury in UC [12,13]. Other dominant intestinal bacteria, such as *Lactobacillus* and *Bacteroides*, are capable of converting dietary Trp into a variety of bioactive compounds [14]. Trp metabolism predominantly proceeds through three pathways: the kynurenine (Kyn) pathway, regulated by indoleamine 2,3-dioxygenase 1 (IDO1) [15]; the indole pathway; and the serotonin (5-HT) pathway [16]. Gut microbiota-dependent Trp metabolism strongly modulates aryl hydrocarbon receptor (AhR) activity, thereby alleviating UC symptoms and preserving intestinal barrier function [7,17]. Multiple Trp metabolites, including indole-3-acetic acid (IAA), kynurenic acid (KYNA), and 5-hydroxyindoleacetic acid (5-HIAA), can activate the AhR pathway and further facilitate intestinal barrier repair [18,19]. In summary, the gut microbiota, Trp metabolism and AhR activity are closely linked in the maintenance of intestinal homeostasis. These observed associations provide novel insights and potential strategies for the therapeutic intervention of UC.

Emerging evidence indicates that dietary supplements targeting gut microbiota to regulate Trp metabolism may effectively ameliorate intestinal barrier dysfunction in UC. Representative agents include Schisandra chinensis pectin polysaccharide, turmeric powder, and β-glucan [20,21,22]. Astragaloside IV (AS-IV) is a principal saponin derived from *Astragalus membranaceus* (Fisch.), a widely used herb in traditional Chinese medicine and functional foods. Its potential therapeutic effects against UC are currently being explored [23]. In preclinical models, AS-IV has been shown to mitigate UC symptoms by modulating macrophage polarization via the STAT signaling pathway [24]. Multiple studies have further demonstrated that AS-IV also exerts protective effects against both DSS-and TNBS-induced colitis, with mechanisms involving the regulation of energy metabolism, inhibition of the PI3K/AKT pathway, and restoration of the Th17/Treg balance [25,26,27,28]. Furthermore, AS-IV may function by modulating the gut microbiota–butyrate metabolic axis, thereby reinforcing intestinal homeostasis [29]. However, whether AS-IV affects intestinal Trp metabolic homeostasis in colitis remains largely unclear.

In the present study, an integrated multi-omics approach, comprising 16S rRNA gene sequencing, untargeted fecal metabolomics, and label-free proteomics, was applied to investigate the protective effects of AS-IV against UC in mouse models. This work focused on host and gut Trp metabolism and aimed to provide multi-omics-based correlative evidence supporting the anti-colitic potential of AS-IV.

## 2. Materials and Methods

### 2.1. Materials

**Figure 1:**
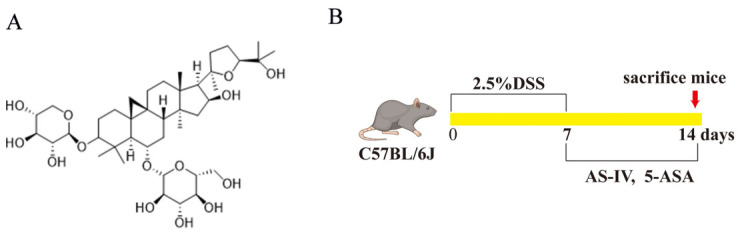
Schematic representation of the experimental design and chemical structure of AS-IV. (**A**) Structural formula of AS-IV. (**B**) Schematic of the animal experimental procedure.

Astragaloside IV (AS-IV, CAS: 84687-43-4, A928102-1g, with a purity of ≥95%) was supplied by Macklin (Shanghai, China), and its chemical structure is depicted in Figure 1A. Colitis-inducing DSS (36–50 kDa) was procured from Dalian Meilun Biotechnology Co., Ltd. (Dalian, China). Luling Pharmaceutical (Jiamusi, China) supplied 5-aminosalicylic acid (5-ASA) used in this study. ELISA kits for TNF-α, IL-6, and IL-1β (catalog nos. F2132-A, F2163-A, F2040-A) were obtained from Fankel Biotechnology (Shanghai, China). Anti-rabbit IgG secondary antibodies were acquired from BOSTER (Wuhan, China). The IDO1 antibody (MU131118S, 1:500) was sourced from Abmart (Shanghai, China). Antibodies for TPH1 (A45018, 1:1000), DDC (A59650, 1:1000), MAO-A (A91506, 1:1000), and AhR (A28618, 1:1000) were acquired from Nature Biosciences (Hangzhou, China). Antibodies against NF-κB p65 and its phosphorylated form (catalog nos. 8242 and 3033, respectively) were obtained from Cell Signaling Technology (Danvers, MA, USA) at a 1:1000 dilution.

### 2.2. Animals

SPF-grade male C57BL/6 mice weighing 18–20 g (6–8 weeks of age) were acquired from Huafukang Biotechnology, Beijing, China, under animal license No. SCXK (Jing) 2024-0003. Mice were allowed a 7-day acclimation period to the laboratory environment prior to experimentation. During this period, all animals were maintained under standard SPF conditions with unrestricted access to food and water. The animal facility was maintained under controlled conditions: a temperature of 22 ± 2 °C, relative humidity ranging from 55% to 60%, and a 12 h light/dark cycle. All animal procedures received approval from the Medical Ethics Committee of Shanxi University of Chinese Medicine (approval number: AWE 202412259).

### 2.3. Animal Experiments

A total of 50 mice were randomized into five groups, with 10 mice in each group, receiving either no treatment (Control), DSS alone (Model), AS-IV at 25 mg/kg (AS-IV-L), AS-IV at 100 mg/kg (AS-IV-H), or 5-ASA at 290 mg/kg (positive control). To induce UC, the animals received drinking water with DSS at a final concentration of 2.5%. AS-IV and 5-ASA were administered via intragastric gavage, and all mice were provided with a standard diet. General health status was assessed daily by monitoring body weight and calculating Disease Activity Index (DAI) scores, as previously described [30]. The experimental setup is depicted in Figure 1B.

### 2.4. Mouse DAI Score

The DAI was assessed based on three common symptoms of UC: weight loss, stool consistency, and rectal bleeding, in accordance with previously established methods [31]. The overall DAI score was calculated as the mean of the three individual indices, as detailed in Table S1.

### 2.5. Histopathological, Histochemical and Immunohistochemical Assessment

The above assessments were carried out as previously described [32]. Following overnight fixation in 4% paraformaldehyde, colonic tissues were processed for dehydration and cut into 4-μm sections. For histopathological examination, the colonic sections were stained with hematoxyl and eosin (HE) and Alcian Blue Periodic Acid Schiff (AB-PAS). Tissue sections for Immunohistochemical (IHC) were incubated overnight at 4 °C with primary antibodies against Claudin-1, Occludin, ZO-1, and 5-HT. This was followed by a 30 min incubation with secondary antibodies at 37 °C after three washes with cold phosphate-buffered saline (PBS). Images were captured using a digital slide scanner, and ImageJ 1.52a software was employed to quantify AB-PAS staining and IHC expression levels of the target proteins Mean optical density (MOD) was calculated as integrated optical density (IOD) divided by the area of positive pixels. MOD represents the average intensity of positive staining signals and is widely used to assess the positive expression level based on staining intensity.

### 2.6. Genomic DNA Extraction and 16S-rRNA Sequencing of Feces

The extraction of DNA from fecal samples (100–200 mg) was conducted using the QIAamp^®^ PowerFecal^®^ Pro DNA Kit (QIAGEN, Hilden, Germany). Initially, the samples were homogenized with glass beads to mechanically disrupt bacterial cell walls, in Buffer ATL (QIAGEN) supplemented with 10% PVP to remove PCR inhibitors (e.g., humic acids and polyphenols) from fecal samples. The homogenate was then incubated at 65 °C for 20 min to enhance cell lysis, activate proteinase K, and inactivate endogenous nucleases, followed by centrifugation to separate the DNA-containing supernatant from cellular debris and bound inhibitors. The supernatant obtained from this step was further processed with magnetic beads utilizing the Kingfisher instrument (Thermo, Waltham, MA, USA), and the extracted DNA was subsequently stored. Amplification of the V3V4 region of bacterial 16S rDNA was performed using Phanta Max Master Mix polymerase and primers 338F and 806R in a 50 μL PCR reaction under specified conditions. The PCR products were then purified, denatured, circularized, and linear DNA was digested. DNA nano balls (DNBs) were generated by amplifying the circular library through phi29 and rolling circle amplification. Sequencing of the DNBs was carried out using the DNBSEQ-G400 platform, producing paired-end reads of 300 bases. Library construction and DNBSEQ sequencing were performed according to a previously established protocol [33].

### 2.7. LC-MS-Based Untargeted Metabolomics Analysis

Untargeted metabolomics profiling was carried out following the workflow detailed in [34]. A fecal sample (20 mg) was mixed with methanol/water (7:3, *v*/*v*, 400 μL) containing an internal standard. The sample was subjected to vortexing for 3 min (3 min), followed by sonication in an ice bath for 10 min, and an additional 1 min vortexing. Afterward, the sample was maintained at −20 °C for 30 min. Following centrifugation at 4 °C and 12,000 rpm for 10 min, the precipitate was removed. The supernatant underwent a second centrifugation for 3 min, and 200 μL aliquots were prepared for liquid chromatography-mass spectrometry (LC-MS) analysis.

The initial LC-MS method employed a T3 column (Waters ACQUITY Premier HSS T3 Column 1.8 μm, 2.1 mm × 100 mm) under positive ionization conditions, utilizing a gradient elution of 0.1% formic acid in water and acetonitrile. Chromatographic separation was performed using a gradient elution program: starting at 5%, increasing to 20% in 2 min, 60% in 3 min, and 100% in 1 min, followed by a 1.5 min hold at 100% and a 2.4 min re-equilibration at 5%. The column temperature was kept constant at 40 °C, and the mobile phase was delivered at 0.4 mL/min, with a sample injection volume of 4 μL.The second method applied the same gradient under negative ionization conditions.

Mass spectrometry (MS) analyses (Q Exactive HF-X, Thermo) included full-scan MS at a resolution of 35,000 and data-dependent tandem MS (MS/MS) scans with dynamic exclusion over the mass-to-charge ratio (*m*/*z*) range of 75–1000, employing electrospray ionization in both ionization modes. Additional MS settings are shown in the attached Table S2. The raw mass spectrometry data were converted to mzML format using ProteoWizard, followed by peak picking, alignment, and retention time correction with the XCMS program, the detailed data processing pipeline is provided in Table S3.

### 2.8. Label-Free Quantitative Proteomics

Proteomic analyses were performed according to previously described protocols [35,36], with slight modifications in this study. Mouse colonic tissues were proteomically analyzed. Frozen samples were pulverized in cold T-PERTM reagent (78510, Thermo), and protein levels were measured using a BCA assay. Each group provided 150 μg samples, reduced with DTT at 37 °C for 2.5 h, and alkylated with IAA at room temperature, Trypsin digestion occurred at 37 °C for 20 h. Peptides were acidified, desalted with C18 ZipTip, and reconstituted in 0.1% formic acid for HPLC-MS/MS analysis. A 5 μg tryptic digest was processed using an EASY-nLC1200 nano-HPLC system with 0.1% formic acid and 80% acetonitrile/20% formic acid mobile phases. Samples were enriched, desalted, and separated at 300 nL/min over a 120 min gradient. Analysis was done on a positive-ion Orbitrap Exploris 240 (Thermo) over a 350–1500 *m*/*z* range, with one full MS scan and 20 MS2 scans acquired per cycle. Additional MS parameters are provided in Table S4. Data were quantified label-free using Proteome DiscovererTM 2.5 (Thermo) and matched to the International Protein Index via the UniProt_mouse_And_model_organism_1009_2024_07_10 database, with a 10 ppm precursor mass tolerance, 0.02 Da fragment mass tolerance, and allowance for two missed cleavages. Fixed carbamidomethylation and tryptic digestion were applied, with a 0.01 FDR for data filtering.

### 2.9. Quantitative Real-Time PCR Analysis

Total RNA extraction and reverse transcription were performed as previously described with minor modifications [37]. Colonic tissues were utilized for the extraction of total RNA using the TRIzol reagent. The extracted RNA was subsequently reverse-transcribed into complementary DNA (cDNA) employing the PrimeScript™ RT kit (Takara, Beijing, China). Quantitative polymerase chain reaction (qPCR) was conducted using the TB Green^®^ Premix Ex Taq™ II (Takara, Beijing, China) on a QuantStudio™ platform (Applied Biosystems, Thermo ). All primers, detailed in Table S5, were designed and synthesized by Sangon Biotech (Shanghai, China). The amplification protocol consisted of an initial denaturation at 95 °C for 30 s, followed by 40 cycles of 95 °C for 3 s and 60 °C for 30 s, concluding with a melting curve analysis. The relative expression levels of target gene mRNA were quantified using the 2^−ΔΔCt^ method, with GAPDH serving as the reference housekeeping gene.

### 2.10. Western Blot

Colonic tissues were lysed in RIPA buffer, and protein concentrations were measured with a BCA assay kit. Then, 30 μg of each protein sample was separated by SDS-PAGE and transferred to PVDF membranes. The membranes were blocked with 5% BSA for 3 h, incubated with primary antibodies overnight at 4 °C, washed with TBST, and incubated with secondary antibodies for 1 h at room temperature. Protein bands were visualized with ECL reagent and analyzed with ImageJ software. The procedures were performed as previously described [38].

### 2.11. Statistical Analysis

Data are shown as mean ± SD. Statistical analyses used GraphPad Prism 10. One-way ANOVA with Tukey’s post hoc test compared multiple groups. Spearman’s rank correlation analyzed links between inflammatory cytokines, dominant gut microbiota, and key metabolites. A *p*-value < 0.05 indicated statistical significance.

## 3. Results

### 3.1. AS-IV Alleviated Intestinal Injury Induced by DSS in UC Mice

**Figure 2:**
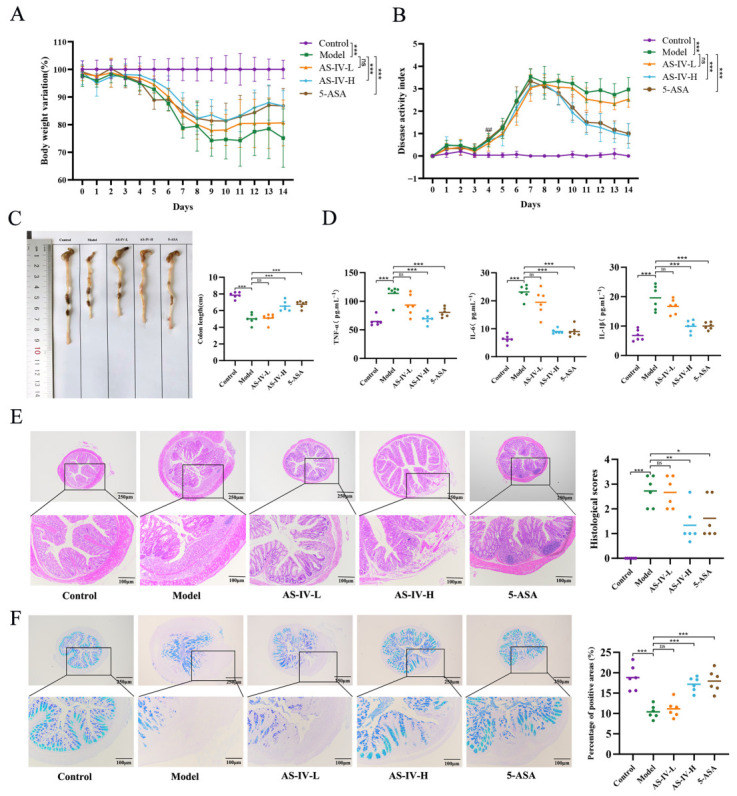
AS-IV alleviated intestinal injury induced by DSS in UC mice. (**A**) Body weight profiles. (**B**) DAI profiles. (**C**) Representative macroscopic photographs of colonic tissues. (**D**) ELISA analysis of serum TNF-α, IL-6, and IL-1β levels. (**E**) Histopathological changes in colonic sections (H&E staining). (**F**) Mucin secretion (AB-PAS staining). *p <* 0.05 (*), *p <* 0.01 (**), *p <* 0.001 (***); n = 10 for panels (**A**,**B**), n = 6 for panels (**C**–**F**).

To investigate the protective effects of AS-IV against UC, a DSS-induced colitis model was established in mice, as previously described [31]. Compared with the control group, DSS-treated mice displayed typical colitis manifestations, such as a marked reduction in body weight, elevated DAI values, and shortened colonic length (Figure 2A–C). Administration of AS-IV effectively mitigated DSS-induced colitis symptoms, with the most pronounced effects observed in the AS-IV-H group. In addition, the serum levels of TNF-α, IL-1β, and IL-6 were higher in the model group, and such upregulation was significantly reversed by treatment with AS-IV-H (Figure 2D). Histological analysis via H&E staining revealed pronounced pathological changes in the model group, characterized by crypt loss, severe injury to the intestinal epithelium, mucosal edema, and infiltration of inflammatory cells, in contrast to the control group (Figure 2E). Intervention with 100 mg/kg of AS-IV significantly mitigated these pathological abnormalities and substantially restored the architecture of colonic crypts. Furthermore, AB-PAS staining demonstrated that DSS administration resulted in significant reductions in colonic mucus production and goblet cell numbers (Figure 2F). Conversely, treatment with AS-IV-H significantly restored goblet cell density and markedly enhanced mucin expression within the colon.

### 3.2. AS-IV Maintained the Colonic Barrier Integrity in DSS-Treated Mice

**Figure 3:**
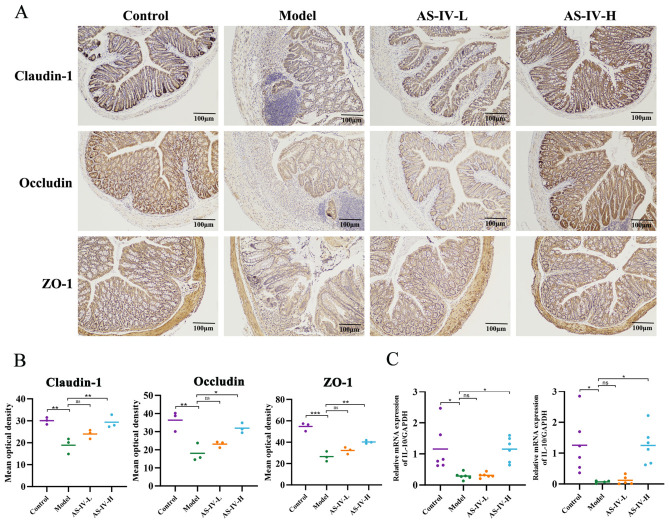
AS-IV maintained the colonic barrier integrity in DSS-treated mice. (**A**,**B**) IHC staining and quantitative analysis of Claudin-1, Occludin, and ZO-1. (**C**) Relative mRNA expression levels of IL-10 and IL-22. *p <* 0.05 (*), *p* < 0.01 (**), *p* < 0.001 (***).

The disruption of intestinal barrier integrity is a typical pathological feature of UC, as reflected by the markedly reduced levels of tight junction proteins. In contrast to the control group, the DSS-induced model displayed a marked depletion of Claudin-1, Occludin, and ZO-1 expression in colonic tissues, as evidenced by immunohistochemical analysis (Figure 3A,B). On the contrary, administration of AS-IV-H markedly reversed the downregulation of these intestinal barrier proteins in the colon of colitis mice. Moreover, the mRNA expression of the critical barrier-protective cytokines IL-10 and IL-22 was significantly enhanced in colon tissue following AS-IV-H intervention (Figure 3C). Collectively, our findings indicate that AS-IV-H intervention effectively mitigated the primary pathological characteristics of DSS-induced colitis and preserved the integrity of the colonic mucosal barrier. Therefore, 100 mg/kg AS-IV was identified as the optimal dose for subsequent experiments.

### 3.3. AS-IV Ameliorated Gut Microbiota Dysbiosis and Increased AKK Abundance

**Figure 4:**
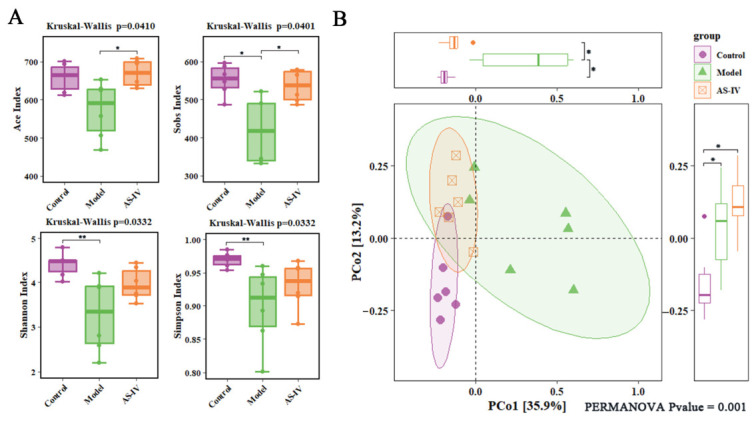
AS-IV rescues gut microbiota in colitis mice. (**A**) Fecal microbiota alpha-diversity assessment (Ace, Sobs, Shannon, and Simpson indexes). (**B**) Principal coordinate analysis (PCoA). *p* < 0.05 (*), *p* < 0.01 (**), Data are from 6 independent samples.

Disturbances of the gut microbial ecosystem are critically implicated in the development and progression of UC. To explore the possible protective effects of AS-IV on DSS-triggered gut microbial dysbiosis, fecal samples were subjected to 16S rRNA gene sequencing to evaluate the diversity and structure of the gut microbiota. Analysis of alpha diversity demonstrated a reduction in the model group, which was ameliorated by AS-IV treatment (Figure 4A). PCoA further illustrated distinct differences in gut microbial community composition across groups (Figure 4B), supporting that AS-IV significantly mitigated DSS-induced gut microbial dysbiosis and reversed its structure toward normal levels.

**Figure 5:**
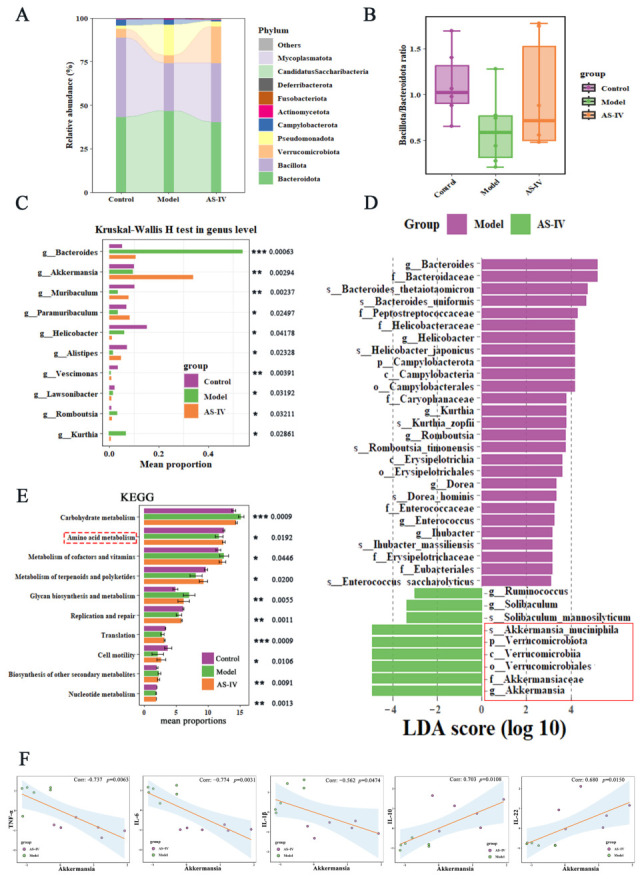
AS-IV mitigated gut microbiota dysbiosis and elevated the relative abundance of AKK. (**A**) Microbial community composition at the phylum level. (**B**) Bacillota/Bacteroidota ratio. (**C**) Genus-level microbial profiles (Kruskal–Wallis test). (**D**) LEfSe analysis of AS-IV vs. Model groups (LDA score > 3, *p* < 0.05). (**E**) Functional prediction of gut microbiota via KEGG pathway enrichment analysis using PICRUSt2. (**F**) Spearman correlation between AKK and inflammatory cytokines. Kruskal–Wallis H test for statistical analysis, *p* < 0.05 (*), *p* < 0.01 (**), *p* < 0.001 (***), Data are from 6 independent samples.

To further elucidate the gut microbiota, we evaluated the taxonomic similarities among the three groups. At the phylum level, the model group exhibited a higher abundance of *Bacteroidota* and *Pseudomonadota* and a lower abundance of *Bacillota* and *Verrucomicrobiota* compared to the control group. AS-IV treatment mitigated these alterations, restoring microbial abundance to levels detected in the control group (Figure 5A). The *Bacillota/Bacteroidota* ratio was reduced in the model group (0.59) versus the control group (1.05), but increased to 0.86 following AS-IV intervention (Figure 5B). At the genus level, AS-IV treatment reversed the reductions in AKK, *Ruminococcus*, *Muribaculum*, and *Alistipes*, as well as the increases in *Bacteroides*, *Romboutsia*, and *Kurthia* observed in the model group (Figure 5C). Furthermore, AKK was identified as the principal discriminatory taxon within the AS-IV group through LEfSe analysis in conjunction with the Wilcoxon test (Figure 5D; Figure S1; LDA > 3, *p* < 0.05). Importantly, Spearman correlation analysis revealed that AKK abundance was negatively correlated with TNF-α, IL-6 and IL-1β, and positively correlated with IL-10 and IL-22, all at statistically significant levels (*p* < 0.05) (Figure 5F). Meanwhile, the KEGG result indicated that AS-IV treatment was significantly associated with pathways related to amino acid metabolism (Figure 5E). Collectively, these findings suggest that AS-IV improves intestinal microbiota dysbiosis, and AKK may play a crucial role in this process.

### 3.4. AS-IV Alleviates DSS-Induced Colitis via Modulating Trp Metabolism

**Figure 6:**
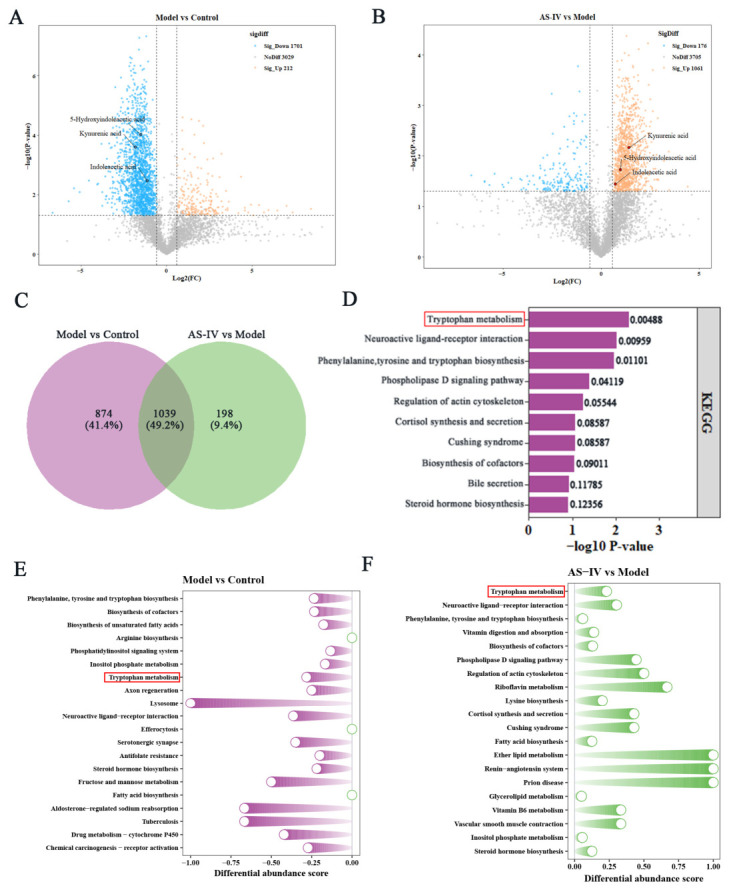
AS-IV modulated the Trp metabolic profiles in the gut. (**A**,**B**) Volcano plots of metabolites between Model vs. Control (**A**) and AS-IV vs. Control (**B**). (**C**) Venn diagram of metabolites. (**D**) KEGG enrichment analysis of metabolites among the three groups. (**E**,**F**) DA scores of Model vs. Control (**E**) and AS-IV vs. Model (**F**). The DA score represents the mean overall change in quantified metabolites in each pathway; a score of 1 indicates overall upregulation of pathway metabolites, and −1 indicates overall downregulation. Data are from 6 independent samples.

**Figure 7:**
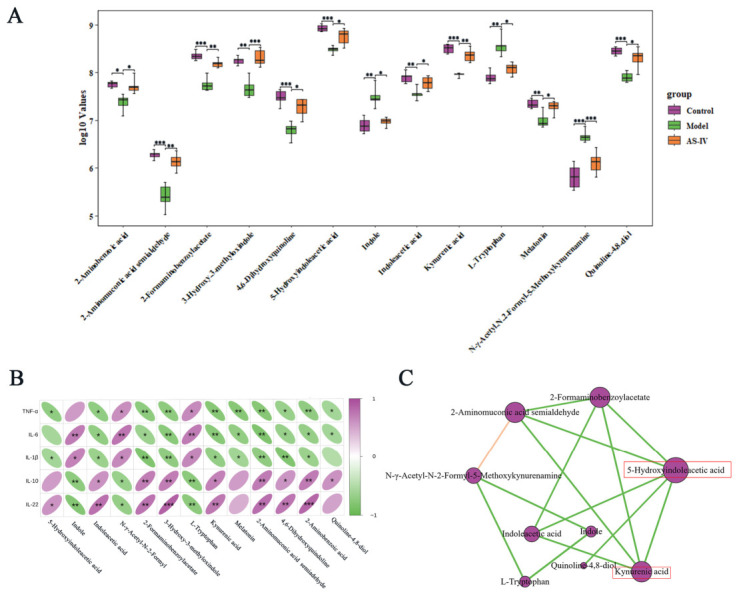
Trp metabolities and their correlation with inflammatory cytokines. (**A**) Relative quantification of Trp metabolites in mouse feces. (**B**) Spearman correlation heatmap of Trp metabolites and inflammatory cytokines. (**C**) Pearson correlation network of Trp metabolites. Green and orange lines represent positive and negative correlations, respectively. Node size corresponds to connectivity (number of significantly correlated substances). Only correlations with coefficients > 0.8 are displayed. *p* < 0.05 (*), *p* < 0.01 (**), *p* < 0.001 (***). Data are from 6 independent samples.

Given the regulatory impact of AS-IV on the gut microbiota, untargeted metabolomics analysis was conducted on fecal samples to identify functional microbial metabolites that may mediate the protective effects of AS-IV against UC. Utilizing metabolomics databases, 4942 metabolites were confirmed at the MS/MS verification level (Table S6). Amino acids and their metabolites represent the most prevalent category across all groups, accounting for 20.7% of the total (Figure S2A). The PCA and OPLS-DA analyses revealed distinct metabolic differences between the model and control groups, which were significantly restored by AS-IV treatment (Figure S2B–G). Differential metabolites were identified according to the following thresholds: a fold-change less than 0.67 or greater than 1.5, a VIP value exceeding 1, a *p* value below 0.05 and a false discovery rate (FDR) of less than 0.2. A comparison between the model and control groups revealed 212 upregulated metabolites and 1701 downregulated metabolites. In contrast, comparisons between the AS-IV and model groups identified 1061 elevated and 176 reduced metabolites (Figure 6A,B). Based on the differentially expressed metabolites (DEMs) identified, Trp metabolism was recognized as the most significantly altered pathway by KEGG pathway enrichment analysis (Figure 6C,D). Differential abundance (DA) analysis revealed that the majority of Trp metabolism DEMs were significantly downregulated in the model group compared with the control groups, but exhibited an increase following AS-IV administration (Figure 6E,F). As shown in Figure 7A, levels of the 13 Trp metabolites (including 5-HIAA, KYNA, and IAA) were markedly altered in the model group relative to the control group, and these concentrations were significantly reversed following AS-IV administration. Spearman correlation analysis (Figure 7B) demonstrated that these 13 Trp metabolites were correlated with certain pro-inflammatory and anti-inflammatory cytokines. Pearson correlation analysis identified 5-HIAA and KYNA as key differential metabolites that may underlie the beneficial effects of AS-IV against UC (Figure 7C). In summary, the protective effect of AS-IV against UC are associated with the regulation of Trp metabolism and the restoration of key metabolites including 5-HIAA and KYNA (authentic standard MS/MS spectra shown in Figure S3).

### 3.5. AS-IV Regulated Trp Metabolism in Host–Gut Microbiota Co-Metabolism

**Figure 8:**
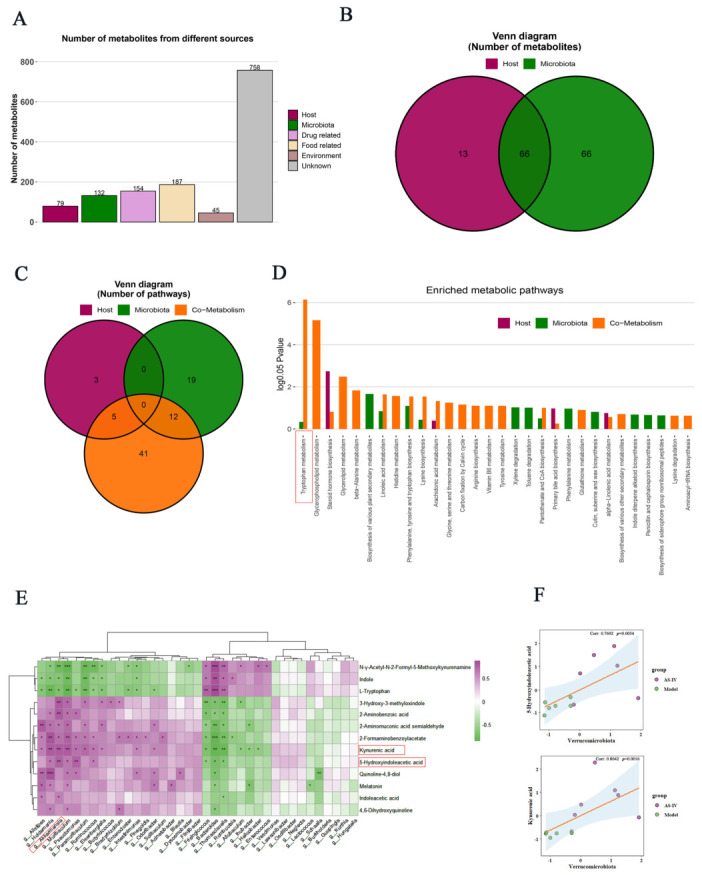
AS-IV regulated Trp metabolism in host–gut microbiota co-metabolism. (**A**) Classification and traceability of differentially abundant metabolites. (**B**) Venn analysis of metabolites from host and microbial origin. (**C**) Venn diagram of enriched metabolic pathways by origin-based MPEA. (**D**) Enriched metabolic pathways visualized from origin-specific MPEA. (**E**) Spearman correlation heatmap between genus-level microbial taxa and fecal metabolic profiles. (**F**) Spearman correlation analysis between Verrucomicrobiota and Trp metabolites (5-HIAA and KYNA). *p* < 0.05 (*), *p* < 0.01 (**), *p* < 0.001 (***), Data are from 6 independent samples.

The host–microbe co-metabolism system is integral to the pathophysiology of UC [39]. Our experimental findings indicate that AS-IV exerts a significant impact on the composition of gut microbiota and the fecal untargeted metabolic profile. To elucidate the mechanistic underpinnings of AS-IV’s protective effects, we utilized the MetOrigin2.0 platform to assess the source attribution of differential metabolites and gut microbiota across various experimental groups [40]. Out of the 597 metabolites identified, 66 were co-metabolites produced by both the host and gut microbiota, 66 were exclusive to the microbiota, and 13 were specific to the host (Figure 8A,B, Table S7). Through origin-based metabolic pathway enrichment analysis (MPEA), we identified 8, 31, and 58 pathways associated with the host, microbiota, and co-metabolism databases, respectively (Figure 8C, Tables S8–S10). Trp metabolism was the most significantly enriched pathway in the co-metabolic pathway (Figure 8D). To explore the associations between gut microbiota and fecal metabolites, Spearman correlation analysis was employed to identify bacterial taxa involved in Trp metabolism (Figure 8E). The analysis revealed that the bacterial taxa *Alistipes*, *Muribaculum*, and AKK exhibited significant associations with at least eight metabolites involved in Trp metabolism. In contrast, *Flintibacter*, *Vescimonas*, *Lawsonibacter*, *Oscillibacter*, and *Neglecta* did not demonstrate such associations, this may be attributed to their relatively low abundance and weak response to the intervention. The Trp metabolites 5-HIAA and KYNA were positively correlated with the relative abundance of AKK. Interestingly, *Verrucomicrobiota*, the phylum to which AKK belongs, displayed a consistent correlation trend (Figure 8F). Together, our results indicate that the beneficial effects of AS-IV against UC are associated with the modulation of Trp metabolism in both the host and gut microbiota. Multiple key bacterial genera were closely correlated with Trp metabolic alterations, among which AKK showed a potential association with Trp-related metabolites (5-HIAA and KYNA).

### 3.6. AS-IV Regulated the Expression of Key Enzymes in Colonic Trp Metabolism

**Figure 9:**
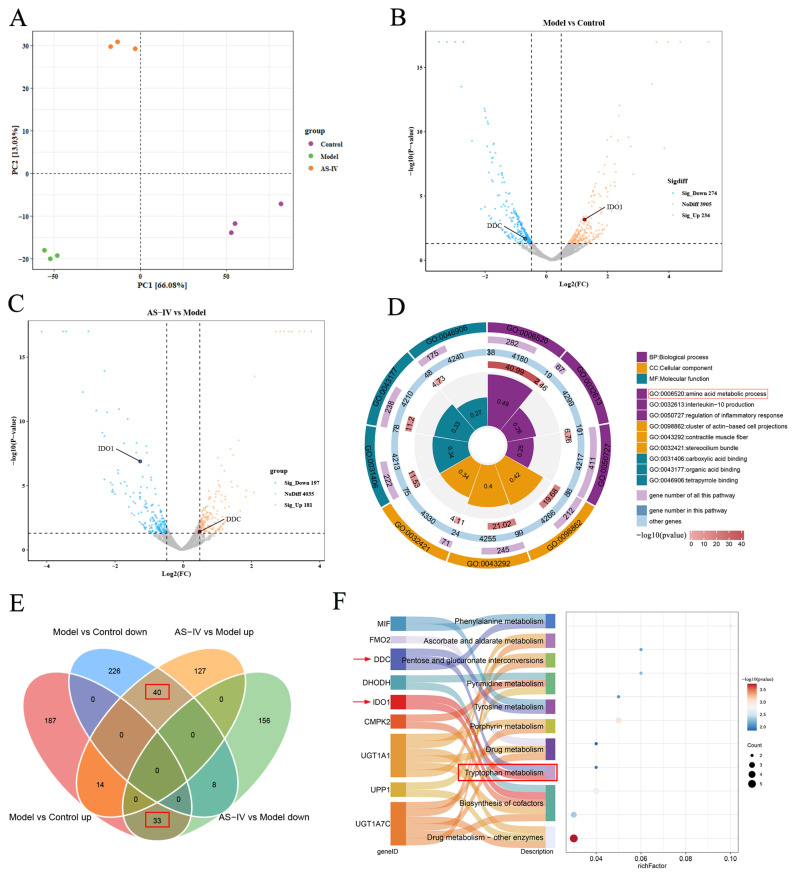
AS-IV induced extensive alterations in protein expression in mouse colonic tissue. (**A**) PCA score plots of the three experimental groups. (**B**,**C**) Volcano plots of DEPs between Model and Control (**B**) and between AS-IV and Model (**C**). (**D**) GO enrichment analysis of DEPs. (**E**) Venn diagram of DEP overlap. (**F**) KEGG pathway enrichment analysis of DEPs Data were obtained from 3 independent replicates.

To further explore the underlying mechanism, we performed label-free proteomic analysis of colon tissue. Distinct separation of the model, AS-IV, and control groups was visualized by PCA. Along the first principal component (PC1), the AS-IV group clustered between the model and control groups, indicating a partial reversal of the proteomic shift induced by colitis (Figure 9A). We identified a total of 4413 non-redundant proteins, each characterized by at least one unique peptide. Proteins exhibiting a fold change (FC) > 1.4 or FC < 0.71 and *p*-value < 0.05, were classified as differentially expressed proteins (DEPs). Compared with the control group, the model group exhibited 234 upregulated and 274 downregulated proteins. In contrast, the AS-IV group showed 181 upregulated and 197 downregulated proteins when benchmarked against the model group (Figure 9B,C), with 73 of these displaying pronounced expression normalization (Figure 9E, Table S11). Subsequently, we conducted an enrichment analysis on these 73 DEPs. GO enrichment analysis showed significant enrichment of the amino acid metabolic process, in line with our above results (Figure 9D). Consistently, subsequent KEGG pathway enrichment analysis further indicated that Trp metabolism was significantly enriched among the top 10 significant pathways (Figure 9F). Among the DEPs identified, IDO1 and DDC play pivotal roles in Trp metabolism (Figure 9F). IDO1 dominates Trp catabolism through the kynurenine pathway, while DDC functions as a key metabolic enzyme that facilitates the decarboxylation of 5-hydroxytryptophan (5-HTP) into 5-HT [15,16]. In this study, IDO1 expression was significantly elevated in the model group (*p* < 0.05) and markedly reduced following treatment with AS-IV (*p* < 0.001). Conversely, DDC expression exhibited an opposite trend (Table S11).

**Figure 10:**
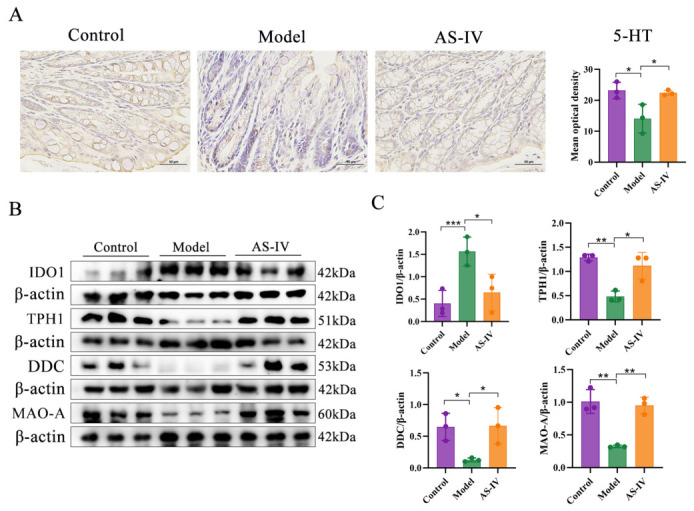
AS-IV regulated the expression of key enzymes in gut Trp metabolism. (**A**) IHC staining of colonic tissue for 5-HT. (**B**,**C**) Colonic expression of IDO1, TPH1, DDC, and MAO-A determined by Western blotting. *p* < 0.05 (*), *p* < 0.01 (**), *p* < 0.001 (***), Data were obtained from 3 independent replicates.

Furthermore, immunohistochemical analysis demonstrated a reduction in 5-HT expression in the DSS-induced UC, which was restored by AS-IV intervention (*p* < 0.05; Figure 10A). Given the critical role of IDO1, DDC, tryptophan hydroxylase 1 (TPH1), and monoamine oxidase A (MAO-A) in regulating Trp metabolism, we employed Western blotting to evaluate whether their expression was altered by AS-IV treatment. The results showed that IDO1 was markedly upregulated in the model group, and this increase was significantly reversed by AS-IV (*p* < 0.05). In contrast, the expression of TPH1, DDC, and MAO-A was significantly decreased in the model group, and these levels were markedly recovered after AS-IV treatment (*p* < 0.05; Figure 10B,C). In summary, AS-IV modulates colonic Trp metabolism primarily by regulating the expression of rate-limiting metabolic enzymes.

### 3.7. AS-IV Alters Colonic AhR Expression and Phosphorylation of NF-κB p65

**Figure 11:**
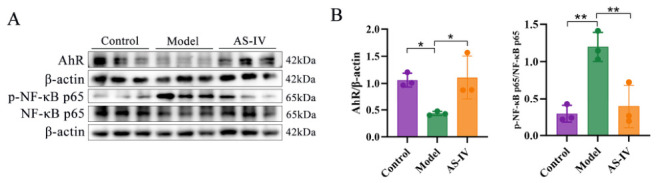
AS-IV exert Intestinal Anti-Inflammatory Effects via AhR/NF-κB p65 axis. (**A**,**B**) Colonic expression of AhR, p-NF-κB p65, and NF-κB p65 determined by Western blotting. *p* < 0.05 (*), *p* < 0.01 (**), Data were obtained from 3 independent replicates.

AS-IV administration altered the levels of Trp metabolites (5-HIAA and KYNA), which have been reported to act as putative AhR ligands and may be linked to the regulation of the NF-κB pathway in previous studies [8,41,42,43]. We therefore performed Western blot analysis to examine the protein levels of AhR and phosphorylated NF-κB p65 in mouse colon tissues. As shown in Figure 11A,B, AS-IV rescued DSS-suppressed AhR expression and reduced aberrant NF-κB p65 phosphorylation in colon tissues.

## 4. Discussion

Ulcerative colitis (UC) is a chronic intestinal inflammatory disorder closely associated with gut dysbiosis and disrupted microbial tryptophan metabolism, which collectively impair intestinal barrier function and aggravate disease progression [6,7,32]. In light of the limited effective therapeutic options currently available for UC, there is an increasing focus on identifying safe and functional alternatives derived from resources with both medicinal and nutritional applications [43,44,45]. AS-IV, the primary active component of *Astragalus membranaceus* (Huangqi), has been traditionally utilized as both a medicinal agent and a dietary supplement [23]. Previous studies have indicated that AS-IV exhibits therapeutic potential against UC, likely linked to beneficial shifts in intestinal microbiota homeostasis and immune responses [23,24,25,26,27,28]. However, how AS-IV relates to host–gut tryptophan metabolic balance during colitis protection is still not fully elucidated.

This work demonstrates that AS-IV ameliorates colonic inflammation and preserves intestinal barrier homeostasis. These protective effects are coupled with marked changes in host–gut Trp metabolism, alongside compositional alterations in gut microbiota, with a significantly higher relative abundance of AKK. In colonic tissue, colitis led to abnormal IDO1 elevation and reduced TPH1, DDC, and MAO-A levels. AS-IV treatment attenuated these aberrant enzyme changes. Meanwhile, colonic AhR expression and NF-κB p65 phosphorylation also presented distinct alterations after AS-IV treatment. Taken together, these data indicate the potential molecular basis underlying the protective effects of AS-IV, supporting its potential as a dietary approach for UC management.

Our 16S rRNA gene sequencing data indicated that AS-IV administration altered the gut microbial community structure in DSS-colitis mice. We observed a significant increase in the relative abundance of AKK, a mucin-degrading bacterium closely linked to intestinal homeostasis. As previously reported, AKK tends to decrease obviously diminished in patients with UC as well as in pertinent experimental models [12,13], and its increased abundance is correlated with ameliorated clinical symptoms in UC patients [46]. Spearman’s correlation analysis revealed an inverse relationship between the enriched population of AKK and the levels of TNF-α, IL-6, and IL-1β, while demonstrating a positive correlation with the IL-10 and IL-22. Furthermore, KEGG pathway enrichment analysis indicated that AS-IV treatment may be closely related to amino acid metabolism-related pathways. These collective observations suggest that AS-IV exerts protective effects against colitis accompanied by gut microbial shifts, elevated AKK abundance, and altered amino acid metabolism.

Consistent with such trends, our untargeted fecal metabolomics analysis further identified Trp metabolism as the most significantly altered pathway within amino acid metabolism following AS-IV intervention. Trp metabolism is closely implicated in intestinal inflammation related to UC [32,47]. Our results revealed that treatment with AS-IV partially restored the fecal levels of 13 key Trp metabolites, including 5-HIAA, KYNA, and IAA, in DSS-induced UC. Among these metabolites, 5-HIAA serves as a critical marker of 5-HT pathway activity, which is essential for maintaining intestinal homeostasis [42]. Dysregulation of 5-HT signaling has been linked to various intestinal complications in patients with UC [15,48,49]. KYNA, an anti-inflammatory metabolite produced via the kynurenine pathway, is strongly associated with disease severity in IBD [19,50]. IAA, a microbiota-derived Trp metabolite, contributes to enhancing intestinal barrier integrity and suppressing excessive inflammatory responses [41,51]. Our data indicate that the beneficial metabolites among the 13 key tryptophan metabolites exhibited a significant negative correlation with pro-inflammatory cytokines, while showing a positive correlation with anti-inflammatory mediators. Pearson correlation analysis further supported that 5-HIAA and KYNA may represent important effector metabolites responsible for the beneficial effects of AS-IV against UC. This evidence substantiates the hypothesis that modulation of Trp metabolism, particularly the levels of 5-HIAA and KYNA, is crucial for mediating the protective effects of AS-IV against UC. Host–microbe co-metabolic analyses revealed a significant enrichment of Trp metabolism in the shared host–gut microbial metabolic pathways, which generally aligns with our untargeted metabolomics data. Moreover, a positive association was observed between AKK abundance and concentrations of 5-HIAA and KYNA by Spearman correlation analysis, which is in line with previous reports. These correlative findings further indicate that AS-IV improves colonic homeostasis via modulating host–microbial Trp co-metabolism. Alterations in AKK are closely correlated with elevated levels of beneficial Trp-related metabolites.

To further elucidate the regulatory effects of AS-IV on host colonic signaling and functional pathways, label-free quantitative proteomic analysis was conducted on colonic tissues from experimental mice. Proteomic profiling combined with downstream functional annotation demonstrated that Trp metabolic pathways represented some of the most significantly altered biological processes upon AS-IV intervention. Additionally, several key differentially expressed proteins involved in Trp metabolism were identified, with IDO1 and DDC exhibiting the most notable changes. IDO1, the key regulatory enzyme of the kynurenine pathway, drives metabolic reprogramming of Trp metabolism. Its overexpression directs the Trp-kynurenine pathway to the pro-inflammatory Quinolinic acid (QA) branch, whereas IDO1 inhibition promotes the anti-inflammatory KYNA pathway and increases KYNA accumulation [52,53,54,55]. In UC, excessive IDO1 activity disturbs QA/KYNA homeostasis, accelerates Trp catabolism, restricts the substrate supply for 5-HT biosynthesis, and exacerbates intestinal mucosal inflammation [15,56,57]. In contrast, DDC is a central enzyme in the 5-HT biosynthetic pathway. Reduced expression of DDC results in inadequate production of 5-HT, which compromises intestinal barrier integrity and aggravates colonic inflammation [51,58,59]. Our proteomic analysis demonstrated that AS-IV downregulated IDO1 expression while upregulating DDC expression in colonic tissues. The dysregulated expression of IDO1 and DDC indicated that AS-IV might regulate the balance between the kynurenine pathway and 5-HT synthesis in colonic tissues.

Immunohistochemical analysis further validated that colonic 5-HT expression was significantly reduced in DSS-induced colitis, and this reduction was markedly attenuated by AS-IV treatment. We also investigated two key enzymes involved in 5-HT homeostasis: TPH1 and MAO-A. TPH1 catalyzes the initial step of 5-HT synthesis, whereas MAO-A facilitates 5-HT degradation [16]. Western blot results demonstrated that AS-IV modulated the expression of TPH1, DDC, and MAO-A, while downregulating IDO1. Combined proteomic and fecal metabolomic data indicated that AS-IV remodels intestinal Trp co-metabolic profiles, accompanied by the regulation of key Trp-metabolizing enzymes in colonic tissues. This modulation towards anti-inflammatory Trp metabolic patterns is associated with reduced colonic mucosal inflammation and better intestinal homeostasis, providing valuable insight into the protective effects of AS-IV against UC.

The AhR is highly expressed in intestinal epithelial cells, where it plays an essential role in modulating host responses to microbial stimuli [60,61]. In patients with UC, AhR expression is diminished, and the absence of AhR exacerbates DSS-induced colitis in mouse models [62,63]. The intake of AhR ligands has been shown to alleviate UC by reducing inflammation [64]. Additionally, NF-κB p65 is hyperactivated in the colonic epithelial cells and macrophages of UC patients, underscoring its critical role in impaired intestinal barrier function and immune dysregulation [65,66]. In our study, AS-IV treatment significantly upregulated AhR expression and suppressed phosphorylation of NF-κB p65 in the colon. In summary, our data demonstrate that the protective effects of AS-IV against UC are associated with altered expression of AhR and reduced p65 phosphorylation.

Our data indicate that the protective effects of AS-IV against colitis are associated with the regulation of gut microbial Trp metabolism. Since this study focuses on omics-based characteristic screening and phenotypic observation, all molecular and microbial alterations cannot be directly defined as causal relationships. In the absence of targeted functional interventions, such as Trp metabolite supplementation and gut microbiota depletion, it remains difficult to validate the direct causal links among these key events. Further pharmacological interventions and in-depth mechanistic studies will be conducted in our future work to confirm this regulatory axis.

## 5. Conclusions

In summary, the present study confirms the protective effect of AS-IV against DSS-induced colitis. Based on multi-omics combined analysis, the current study preliminarily identified that tryptophan metabolism serves as a core functional pathway responding to AS-IV treatment. This therapeutic effect is associated with gut microbiota alteration, particularly the enrichment of AKK, regulation of host–gut Trp metabolism, modulation of AhR expression, and inhibition of NF-κB p65 phosphorylation. These findings shed new light on potential pathways underlying the anti-colitic effects of AS-IV and support the promising development of Astragalus-based functional foods for the intervention of UC.

## Data Availability

The original contributions presented in this study are included in the article/Supplementary Materials. Further inquiries can be directed to the corresponding authors.

## Supplementary Materials

The following supporting information can be downloaded at: https://www.mdpi.com/article/10.3390/foods15101644/s1, Table S1: DAI score; Table S2: Mass spectrometry parameters for Q Exactive HF-X; Table S3: Preprocessing workflow of untargeted metabolomics data; Table S4: Mass spectrometry parameters for Orbitrap Exploris 240; Table S5: Primer sequences of related genes; Table S6: All identified metabolites information; Table S7: Metabolite_Co-Metabolism; Table S8: MPEA_Results_Host; Table S9: MPEA_Results_Microbiota; Tabel S10: MPEA_Results_Co-Metabolism; Table S11: Proteins significantly altered in model vs control and reversed by AS-IV treatment; Figure S1: Phylogenetic tree of the analyzed bacterial strains; Figure S2: AS-IV influenced tryptophan metabolism in the gut with DSS-induced colitis; Figure S3: MS/MS spectrum comparison of the 5-HIAA and KYNA with the authentic standard.
