# Supplementary material for: Astragaloside IV Alleviates DSS-Induced Ulcerative Colitis by Modulating Host–Gut Tryptophan Metabolism
Source: Foods. 2026 May 8;15(10):1644. doi: 10.3390/foods15101644 (PMC13206269; doi:10.3390/foods15101644)
Supplement: Supplementary file 1 [file foods-15-01644-s001.zip › Supplementary Information.pdf]

Supplementary Information

Table S1 DAI score

Score	Weight loss (%)	Stool consistency	Blood in stool
0	None	Normal feces	No blood
1	1%-5%	Loose stool	—
2	6%-10%	Watery diarrhea	Hemoccult
3	11%-15%	Slimy diarrhea	—
4	>15%	Severe watery diarrhea	Gross bleeding

Table S2 Mass spectrometry parameters for Q Exactive HF-X

Parameter	Description	ESI+	ESI-
Spray Voltage	Ionization voltage (V)	3500	3200
Sheath gas (Arb)	Sheath gas flow rate (Arb)	30	30
Aux gas (Arb)	Auxiliary gas flow rate (Arb)	5	5
Ion transfer tube temperature	Ion transfer tube temperature (℃)	320	320
Vaporizer temperature	Vaporizer temperature (℃)	300	300
Scan Range (MS1)	MS1 mass scan range (Da)	75-1000	75-1000
Resolution (MS1)	MS1 resolution	35000	35000
AGC target (MS1)	MS1 automatic gain control target	1E + 06	1E+06
Scan Range (MS2)	MS2 mass scan range (Da)	75-1000	75-1000
Resolution (MS2)	MS2 resolution	17500	17500

Table S3 Preprocessing workflow of untargeted metabolomics data

Step	Operation	Method / Parameters
1. Data Format Conversion	Conversion of raw mass spectrometry data	Using ProteoWizard to convert to .mzML format
2. Peak Picking & Alignment	Peak detection, alignment, and retention time correction	Performed with the XCMS program
3. Missing Value Filtering	Removal of low-quality features	Features with a missing rate > 50% were discarded
4. Missing Value Imputation	Imputation of blank values in remaining features	- Blank rate > 50%: Imputation with 1/5 of the minimum observed value<br>- Blank rate < 50%: Imputation using the KNN algorithm
5. Peak Area Correction	Correction of signal intensities	Performed using the Support Vector Regression (SVR) method
6. Metabolite Annotation	Identification of metabolites	Searched against in-house database, public databases, and the metDNA tool
7. Quality Control Filtering	Selection of high-confidence metabolites	Features with an annotation score ≥0.5 and QC-CV < 0.3 were retained
8. Positive/Negative Mode Merging	Merging features detected in both ionization modes	Retained the entry with the highest annotation confidence and the lowest CV

Table S4 Mass spectrometry parameters for Orbitrap Exploris 240

Parameter	Description	Value
Full MS Resolution	Orbitrap resolution for full scan (MS1)	60,000
MS1 AGC Target	Automatic gain control target for MS1	3×10^6
MS1 Maximum Injection Time	Maximum ion injection time for MS1	50 ms
MS/MS Isolation Window	Precursor ion isolation window for MS2	1.6 Th
MS/MS Resolution	Orbitrap resolution for MS/MS scan (MS2)	15,000
MS/MS AGC Target	Automatic gain control target for MS2	7.5×10^4
MS/MS Maximum Injection Time	Maximum ion injection time for MS2	22 ms
Fragmentation Mode	Ion fragmentation method	HCD
Normalized Collision Energy (NCE)	Normalized collision energy for fragmentation	30 eV

Table S5 Primer sequences of related genes

Gene Name		Primer sequences
GAPDH	Forward	GGTTGTCTCCTGCGACTTCA
	Reverse	TGGTCCAGGGTTTCTTACTCC
IL-10	Forward	ATGCCTGGCTCAGCACTGCT
	Reverse	TTAGCTTTTCATTTTGATCATCATGTA
IL-22	Forward	ATGGCTGTCCTGCAGAAATCTA
	Reverse	TCAGACGCAAGCATTTCTCAGA

Table S6 All identified metabolites information

Comprehensive annotation, classification and basic information of all metabolites identified in the present metabolomic analysis.

Table S7 Metabolite_Co-Metabolism

Co-metabolites derived from host and intestinal microbiota identified and annotated by MetOrigin 2.0.

Table S8 MPEA_Results_Host

MetOrigin 2.0 was used for pathway enrichment analysis of host-derived metabolites. Differentially expressed metabolic pathways involved in host physiological metabolism were summarized and comprehensively analyzed.

Table S9 MPEA_Results_Microbiota

MetOrigin 2.0 was used for pathway enrichment analysis of microbiota-derived metabolites. Differentially expressed metabolic pathways involved in intestinal microbial metabolism were summarized and comprehensively analyzed.

Tabel S10 MPEA_Results_Co-Metabolism

MetOrigin 2.0 was used for pathway enrichment analysis of co-metabolic metabolites from host and microbiota. Differentially expressed metabolic pathways were summarized and comprehensively analyzed.

Table S11 Proteins significantly altered in model vs control and reversed by AS-IV treatment

Differential proteins were screened between the model and normal control groups, and key proteins with expression reversed by AS-IV were summarized.

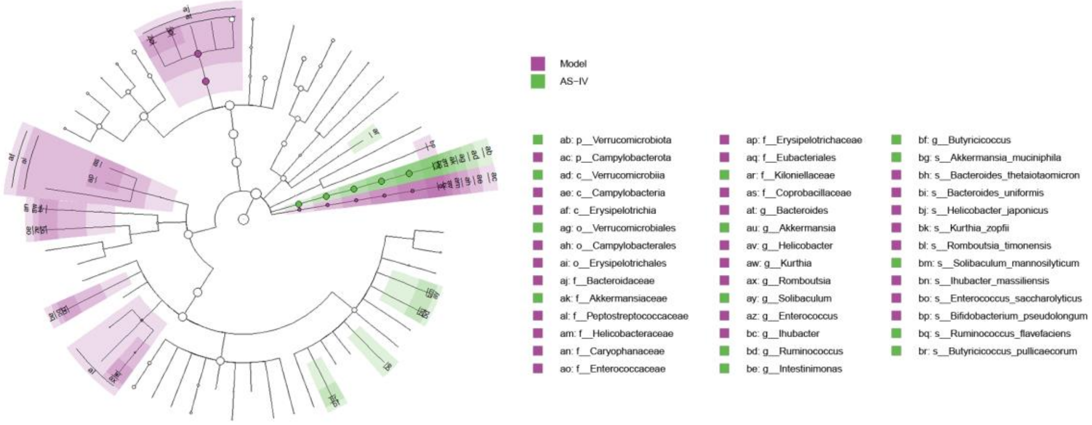

Fig.S1. Phylogenetic tree of the analyzed bacterial strains.

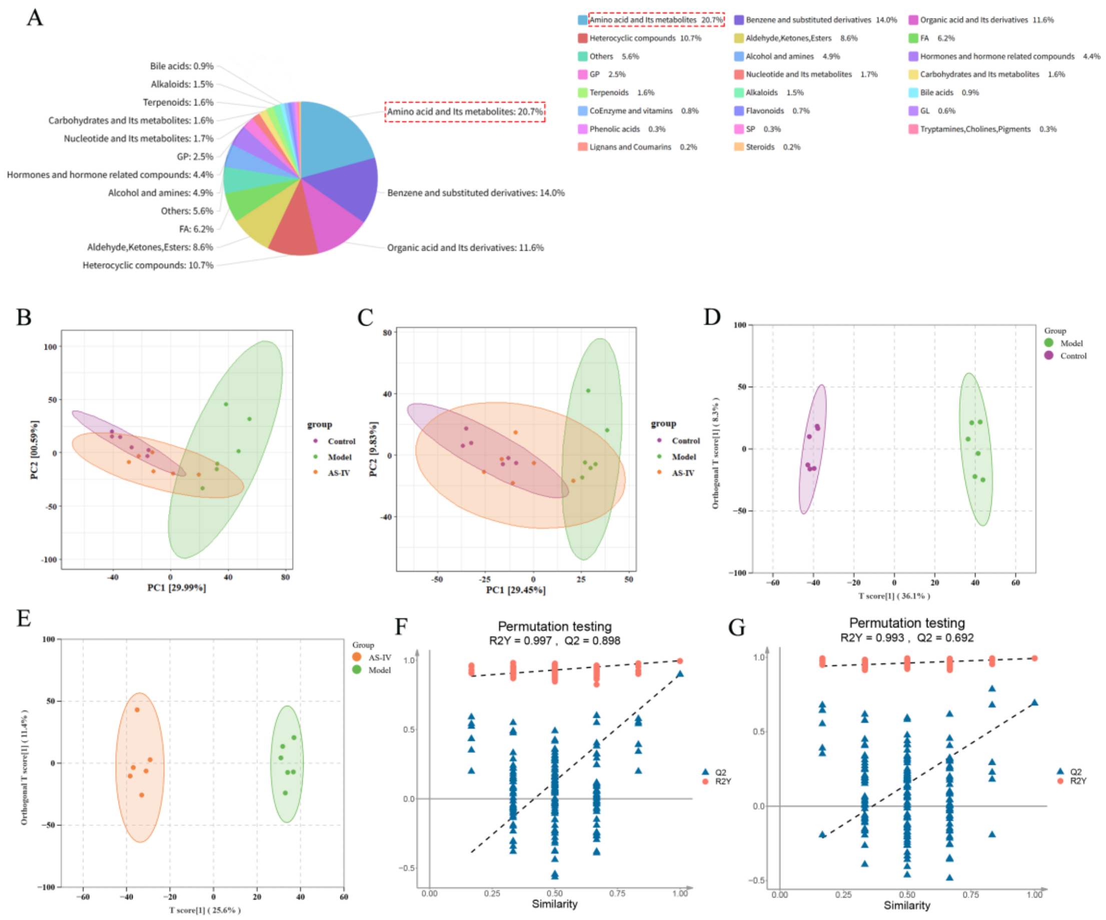

Fig.S2. AS-IV influenced tryptophan metabolism in the gut with DSS-induced colitis

(A)Classifications of metabolites.(B-C) PCA score plots based on three groups in (B) positive and (C)negative ion modes. (D-E) OPLS-DA score plots of Model vs. Control (D) and AS-IV vs. Model (E).(F-G) Permutation test of the OPLS-DA model for Model against Control (F) and AS-IV vs. Model(G)

A

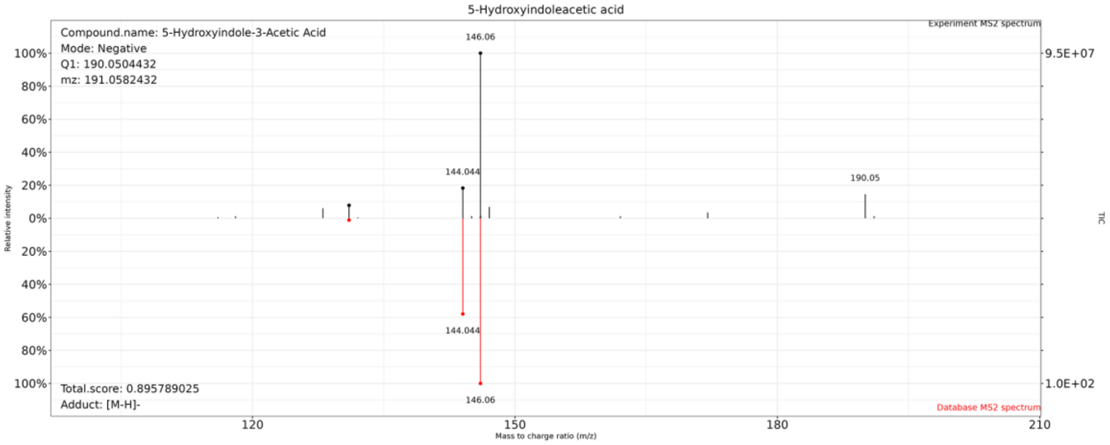

B

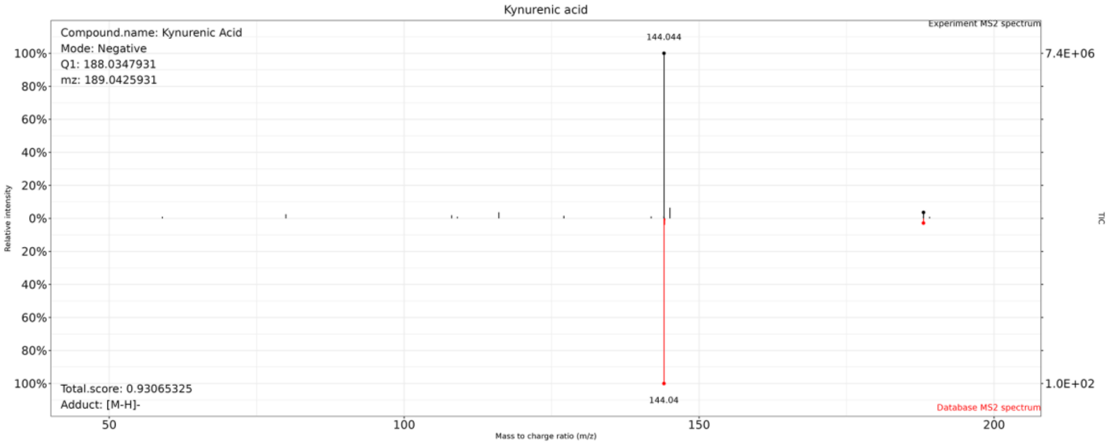

Fig.S3. MS/MS spectrum comparison of the 5-HIAA and KYNA with the authentic standard

(A) MS/MS mirror plot comparison of 5-Hydroxyindoleacetic acid(5-HIAA) with authentic standard.

(B) MS/MS mirror plot comparison of Kynurenic acid (KYNA) with authentic standard.
